# 2-(Furan-2-yl)-5-(2-nitro­benz­yl)-2,3-dihydro-1,5-benzothia­zepin-4(5*H*)-one

**DOI:** 10.1107/S1600536810052098

**Published:** 2010-12-18

**Authors:** Zhao-Hui Huang, Yong Chu, De-Yong Ye

**Affiliations:** a Department of Medicinal Chemistry, School of Pharmacy, Fudan University, Shanghai 201203, People’s Republic of China

## Abstract

The title compound, C_20_H_16_N_2_O_4_S, was prepared by introduction of a 2-nitro­benzyl group to 2-(furan-2-yl)-2,3-dihydro-1,5-benzothia­zepin-4(*5H*)-one *via* an alkaline-catalysed reaction. The thia­zepine ring adopts a twist-boat conformation. The furan ring is oriented at dihedral angles of 56.75 (14) and 10.82 (14)° with respect to the two benzene rings, while the two benzene rings make a dihedral angle of 62.96 (10)°. Weak inter­molecular C—H⋯O hydrogen bonds occur in the crystal structure.

## Related literature

The title compound was prepared as part of an investigation of novel GSK 3β inhibitors. For applications of non-ATP competitive glycogen synthase kinase 3β(GSK 3β) inhibitors, see: Martinez *et al.* (2002 ▶).
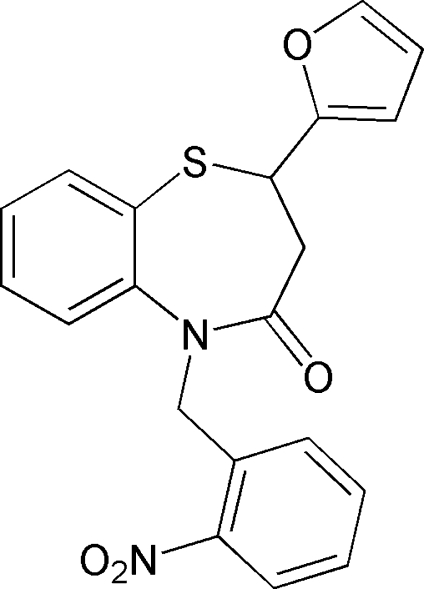


## Experimental

### 

#### Crystal data

C_20_H_16_N_2_O_4_S
*M* *_r_* = 380.41
Monoclinic, 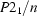

*a* = 11.061 (3) Å
*b* = 8.538 (3) Å
*c* = 19.340 (6) Å
β = 105.535 (4)°
*V* = 1759.7 (9) Å^3^
*Z* = 4
Mo *K*α radiation
μ = 0.21 mm^−1^
*T* = 293 K
0.18 × 0.16 × 0.14 mm

#### Data collection

Bruker SMART CCD area-detector diffractometer
Absorption correction: multi-scan (*SADABS*; Sheldrick, 1996 ▶) *T*_min_ = 0.963, *T*_max_ = 0.971
7699 measured reflections
3458 independent reflections
2755 reflections with *I* > 2σ(*I*)
*R*_int_ = 0.038

#### Refinement

*R*[*F* ^2^ > 2σ(*F* ^2^)] = 0.047
*wR*(*F* ^2^) = 0.140
*S* = 1.10
3458 reflections
244 parameters
12 restraints
H-atom parameters constrained
Δρ_max_ = 0.32 e Å^−3^
Δρ_min_ = −0.34 e Å^−3^

### 

Data collection: *SMART* (Bruker, 2000 ▶); cell refinement: *SAINT* (Bruker, 2000 ▶); data reduction: *SAINT*; program(s) used to solve structure: *SHELXTL* (Sheldrick, 2008 ▶); program(s) used to refine structure: *SHELXTL*; molecular graphics: *SHELXTL*; software used to prepare material for publication: *SHELXTL*.

## Supplementary Material

Crystal structure: contains datablocks I, global. DOI: 10.1107/S1600536810052098/xu5112sup1.cif

Structure factors: contains datablocks I. DOI: 10.1107/S1600536810052098/xu5112Isup2.hkl

Additional supplementary materials:  crystallographic information; 3D view; checkCIF report

## Figures and Tables

**Table 1:** Hydrogen-bond geometry (Å, °)

*D*—H⋯*A*	*D*—H	H⋯*A*	*D*⋯*A*	*D*—H⋯*A*
C2—H2*A*⋯O2^i^	0.93	2.49	3.401 (3)	168
C12—H12*A*⋯O2^ii^	0.93	2.59	3.381 (3)	144

Symmetry codes: (i) 
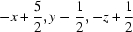
; (ii) 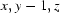.

**Fig. 1.**
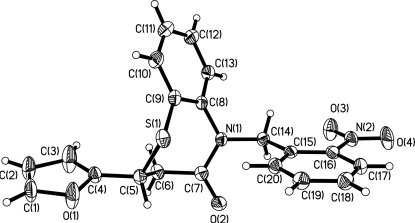
The molecular structure of the title compound showing displacemment ellipsoids at the 50% probability level. H atoms are presented as a small spheres of arbitrary radius.

## supplementary crystallographic information

### Comment

Non-ATP competitive glycogen synthase kinase 3β(GSK 3β) inhibitors might have therapeutic potential for the treatment of diabetes, Alzheimer's disease and cancer (Martinez *et al.*, 2002). The title compound, 5-(2-nitrobenzyl)-2-(furan-2-yl)-2,3-[*b*][1,4]thiazepin-4(5*H*)-one(I), was obtained in our research for novel GSK 3β inhibitors. We report here the crystal structure of the title compound in order to study the relationship between its structure and GSK 3β inhibitory activity.

The molecular structure of the title compound presented on Fig.1. In the crystal structure, the thiazepine ring adopts a similar boat form, while the dihedral angles between the furan ring and the C8>C13, C15>C20 benzene rings are 56.7° and 10.7°, respectively. The dihedral angle between C8>C13 and C15>C20 benzene rings is 63.0°. The crystal packing is stabilized by C—H···O and C—H···N hydrogen bonds (Table 1).

### Experimental

A mixture of 2-(furan-2-yl)-2,3-dihydro-1,5-benzothiazepin-4(5*H*)-one (495 mg, 2 mmol) and 60% sodium hydride (240 mg, 6 mmol) in dry *N*,*N*-dimethylformamide (8 ml) was stirred in ice water bath for 30 minutes, then a solution of 1-(bromomethyl)-2-nitrobenzene (864 mg, 6 mmol) in dry *N*,*N*-Dimethylformamide (6 ml) was added and the resulted mixture was stirred for another 30 minutes. The target compound was extracted by ethyl acetate. After the ethyl acetate evaporated *in vacuo*, the residue was purified by silica gel column chromatography (petroleum ether / ethyl acetate = 10 / 3) to afford 577 mg of (I), yield 76%. Recrystallization from methanol gave light yellow crystals.

### Refinement

All H atoms were placed in the idealized positions with C—H = 0.93–0.97 Å, and with *U*_iso_(H) = 1.2*U*_eq_(C).

### Crystal data

**Table:**

C_20_H_16_N_2_O_4_S	*F*(000) = 792
*M**_r_* = 380.41	*D*_x_ = 1.436 Mg m^−^^3^
Monoclinic, *P*2_1_/*n*	Mo *K*α radiation, λ = 0.71073 Å
Hall symbol: -P 2yn	Cell parameters from 1000 reflections
*a* = 11.061 (3) Å	θ = 2.6–26.7°
*b* = 8.538 (3) Å	µ = 0.21 mm^−^^1^
*c* = 19.340 (6) Å	*T* = 293 K
β = 105.535 (4)°	Block, light-yellow
*V* = 1759.7 (9) Å^3^	0.18 × 0.16 × 0.14 mm
*Z* = 4	

### Data collection

**Table:**

Bruker SMART CCD area-detector diffractometer	3458 independent reflections
Radiation source: fine-focus sealed tube	2755 reflections with *I* > 2σ(*I*)
graphite	*R*_int_ = 0.038
φ and ω scans	θ_max_ = 26.0°, θ_min_ = 1.9°
Absorption correction: multi-scan (*SADABS*; Sheldrick, 1996)	*h* = −13→13
*T*_min_ = 0.963, *T*_max_ = 0.971	*k* = −9→10
7699 measured reflections	*l* = −23→16

### Refinement

**Table:**

Refinement on *F*^2^	Primary atom site location: structure-invariant direct methods
Least-squares matrix: full	Secondary atom site location: difference Fourier map
*R*[*F*^2^ > 2σ(*F*^2^)] = 0.047	Hydrogen site location: inferred from neighbouring sites
*wR*(*F*^2^) = 0.140	H-atom parameters constrained
*S* = 1.10	*w* = 1/[σ^2^(*F*_o_^2^) + (0.0823*P*)^2^ + 0.133*P*] where *P* = (*F*_o_^2^ + 2*F*_c_^2^)/3
3458 reflections	(Δ/σ)_max_ < 0.001
244 parameters	Δρ_max_ = 0.32 e Å^−^^3^
12 restraints	Δρ_min_ = −0.34 e Å^−^^3^

### Special details

**Table:**

Geometry. All e.s.d.'s (except the e.s.d. in the dihedral angle between two l.s. planes) are estimated using the full covariance matrix. The cell e.s.d.'s are taken into account individually in the estimation of e.s.d.'s in distances, angles and torsion angles; correlations between e.s.d.'s in cell parameters are only used when they are defined by crystal symmetry. An approximate (isotropic) treatment of cell e.s.d.'s is used for estimating e.s.d.'s involving l.s. planes.
Refinement. Refinement of *F*^2^ against ALL reflections. The weighted *R*-factor *wR* and goodness of fit *S* are based on *F*^2^, conventional *R*-factors *R* are based on *F*, with *F* set to zero for negative *F*^2^. The threshold expression of *F*^2^ > σ(*F*^2^) is used only for calculating *R*-factors(gt) *etc*. and is not relevant to the choice of reflections for refinement. *R*-factors based on *F*^2^ are statistically about twice as large as those based on *F*, and *R*- factors based on ALL data will be even larger.

### Fractional atomic coordinates and isotropic or equivalent isotropic displacement parameters (Å^2^)

**Table:**

	*x*	*y*	*z*	*U*_iso_*/*U*_eq_	
S1	0.88992 (5)	0.50517 (6)	0.22528 (3)	0.04348 (19)	
N1	0.86748 (14)	0.47987 (17)	0.06247 (8)	0.0344 (4)	
N2	0.54187 (16)	0.5826 (2)	−0.12646 (8)	0.0452 (4)	
O1	1.1607 (2)	0.6320 (2)	0.34025 (9)	0.0879 (7)	
O2	0.95960 (14)	0.70935 (17)	0.05012 (8)	0.0517 (4)	
O3	0.62377 (17)	0.5009 (2)	−0.13854 (8)	0.0672 (5)	
O4	0.44565 (16)	0.6131 (2)	−0.17221 (9)	0.0748 (5)	
C1	1.2646 (3)	0.5769 (3)	0.38975 (14)	0.0791 (9)	
H1A	1.2950	0.6160	0.4360	0.095*	
C2	1.3141 (2)	0.4647 (4)	0.36370 (14)	0.0702 (7)	
H2A	1.3846	0.4070	0.3869	0.084*	
C3	1.2399 (3)	0.4454 (4)	0.29201 (15)	0.0882 (10)	
H3A	1.2542	0.3736	0.2589	0.106*	
C4	1.14695 (19)	0.5473 (2)	0.28057 (10)	0.0419 (5)	
C5	1.04068 (17)	0.5900 (2)	0.21861 (10)	0.0394 (4)	
H5A	1.0320	0.7042	0.2191	0.047*	
C6	1.06808 (18)	0.5463 (2)	0.14781 (10)	0.0408 (5)	
H6A	1.1432	0.6006	0.1442	0.049*	
H6B	1.0844	0.4347	0.1476	0.049*	
C7	0.96163 (17)	0.5866 (2)	0.08346 (10)	0.0383 (4)	
C8	0.86766 (16)	0.3349 (2)	0.09979 (9)	0.0348 (4)	
C9	0.87861 (17)	0.3321 (2)	0.17369 (10)	0.0393 (4)	
C10	0.8751 (2)	0.1872 (3)	0.20661 (12)	0.0523 (5)	
H10A	0.8841	0.1833	0.2558	0.063*	
C11	0.8586 (2)	0.0509 (3)	0.16798 (13)	0.0585 (6)	
H11A	0.8519	−0.0438	0.1904	0.070*	
C12	0.8520 (2)	0.0544 (3)	0.09558 (13)	0.0513 (5)	
H12A	0.8435	−0.0384	0.0695	0.062*	
C13	0.85800 (18)	0.1954 (2)	0.06219 (11)	0.0417 (5)	
H13A	0.8555	0.1970	0.0138	0.050*	
C14	0.77070 (18)	0.5078 (2)	−0.00482 (10)	0.0371 (4)	
H14A	0.7365	0.4077	−0.0245	0.045*	
H14B	0.8098	0.5556	−0.0389	0.045*	
C15	0.66344 (16)	0.6118 (2)	0.00252 (9)	0.0339 (4)	
C16	0.55868 (17)	0.6482 (2)	−0.05424 (9)	0.0368 (4)	
C17	0.46416 (19)	0.7472 (2)	−0.04634 (11)	0.0452 (5)	
H17A	0.3964	0.7693	−0.0854	0.054*	
C18	0.4709 (2)	0.8125 (2)	0.01949 (12)	0.0494 (5)	
H18A	0.4080	0.8794	0.0253	0.059*	
C19	0.5712 (2)	0.7785 (2)	0.07652 (11)	0.0473 (5)	
H19A	0.5761	0.8220	0.1213	0.057*	
C20	0.66531 (18)	0.6798 (2)	0.06802 (10)	0.0407 (4)	
H20A	0.7323	0.6583	0.1076	0.049*	

### Atomic displacement parameters (Å^2^)

**Table:**

	*U*^11^	*U*^22^	*U*^33^	*U*^12^	*U*^13^	*U*^23^
S1	0.0388 (3)	0.0601 (4)	0.0317 (3)	0.0035 (2)	0.0097 (2)	−0.0043 (2)
N1	0.0350 (8)	0.0381 (8)	0.0275 (8)	0.0007 (6)	0.0037 (6)	0.0011 (6)
N2	0.0454 (9)	0.0529 (11)	0.0315 (9)	−0.0075 (8)	0.0002 (7)	0.0006 (7)
O1	0.1060 (14)	0.0885 (13)	0.0440 (9)	0.0542 (11)	−0.0236 (9)	−0.0251 (9)
O2	0.0547 (9)	0.0492 (8)	0.0477 (8)	−0.0111 (7)	0.0076 (7)	0.0070 (7)
O3	0.0678 (11)	0.0909 (13)	0.0368 (9)	0.0228 (9)	0.0033 (8)	−0.0118 (8)
O4	0.0579 (10)	0.1064 (15)	0.0429 (9)	0.0081 (10)	−0.0167 (7)	−0.0144 (9)
C1	0.0895 (19)	0.0787 (18)	0.0430 (13)	0.0247 (16)	−0.0275 (13)	−0.0115 (13)
C2	0.0528 (14)	0.0937 (19)	0.0517 (15)	0.0275 (13)	−0.0073 (11)	−0.0008 (14)
C3	0.0848 (19)	0.110 (2)	0.0542 (15)	0.0532 (18)	−0.0095 (14)	−0.0240 (15)
C4	0.0414 (10)	0.0460 (11)	0.0341 (10)	0.0044 (9)	0.0030 (8)	−0.0063 (8)
C5	0.0375 (10)	0.0409 (10)	0.0371 (10)	0.0039 (8)	0.0053 (8)	−0.0030 (8)
C6	0.0344 (9)	0.0517 (12)	0.0361 (10)	−0.0022 (8)	0.0089 (8)	−0.0026 (9)
C7	0.0385 (10)	0.0431 (11)	0.0343 (10)	−0.0003 (8)	0.0117 (8)	−0.0009 (8)
C8	0.0308 (9)	0.0395 (10)	0.0319 (9)	0.0018 (7)	0.0046 (7)	0.0029 (7)
C9	0.0346 (9)	0.0489 (11)	0.0327 (10)	0.0020 (8)	0.0063 (8)	0.0032 (8)
C10	0.0580 (13)	0.0588 (14)	0.0378 (11)	−0.0007 (11)	0.0092 (10)	0.0130 (10)
C11	0.0672 (15)	0.0487 (13)	0.0548 (14)	0.0000 (11)	0.0081 (12)	0.0148 (11)
C12	0.0561 (13)	0.0403 (11)	0.0521 (13)	0.0026 (10)	0.0051 (10)	0.0026 (9)
C13	0.0421 (10)	0.0447 (11)	0.0364 (10)	0.0030 (8)	0.0073 (8)	0.0008 (8)
C14	0.0409 (10)	0.0398 (10)	0.0283 (9)	0.0005 (8)	0.0051 (8)	−0.0004 (7)
C15	0.0367 (9)	0.0328 (9)	0.0313 (9)	−0.0058 (7)	0.0076 (7)	0.0035 (7)
C16	0.0381 (10)	0.0376 (10)	0.0326 (9)	−0.0074 (8)	0.0058 (8)	0.0015 (7)
C17	0.0363 (10)	0.0487 (12)	0.0455 (12)	0.0000 (9)	0.0023 (9)	0.0044 (9)
C18	0.0438 (11)	0.0492 (12)	0.0552 (13)	0.0075 (9)	0.0131 (10)	−0.0008 (10)
C19	0.0511 (12)	0.0504 (12)	0.0401 (11)	0.0016 (9)	0.0119 (9)	−0.0048 (9)
C20	0.0422 (10)	0.0443 (11)	0.0327 (10)	0.0019 (8)	0.0047 (8)	−0.0008 (8)

### Geometric parameters (Å, °)

**Table:**

S1—C9	1.769 (2)	C8—C13	1.384 (3)
S1—C5	1.854 (2)	C8—C9	1.402 (3)
N1—C7	1.361 (2)	C9—C10	1.396 (3)
N1—C8	1.433 (2)	C10—C11	1.369 (3)
N1—C14	1.466 (2)	C10—H10A	0.9300
N2—O3	1.215 (2)	C11—C12	1.383 (3)
N2—O4	1.216 (2)	C11—H11A	0.9300
N2—C16	1.470 (2)	C12—C13	1.376 (3)
O1—C4	1.336 (3)	C12—H12A	0.9300
O1—C1	1.368 (3)	C13—H13A	0.9300
O2—C7	1.228 (2)	C14—C15	1.518 (3)
C1—C2	1.272 (4)	C14—H14A	0.9700
C1—H1A	0.9300	C14—H14B	0.9700
C2—C3	1.419 (4)	C15—C20	1.389 (3)
C2—H2A	0.9300	C15—C16	1.401 (2)
C3—C4	1.319 (3)	C16—C17	1.384 (3)
C3—H3A	0.9300	C17—C18	1.374 (3)
C4—C5	1.482 (3)	C17—H17A	0.9300
C5—C6	1.526 (3)	C18—C19	1.370 (3)
C5—H5A	0.9800	C18—H18A	0.9300
C6—C7	1.506 (3)	C19—C20	1.382 (3)
C6—H6A	0.9700	C19—H19A	0.9300
C6—H6B	0.9700	C20—H20A	0.9300
			
C9—S1—C5	102.48 (9)	C10—C9—S1	119.28 (15)
C7—N1—C8	122.04 (15)	C8—C9—S1	122.32 (15)
C7—N1—C14	118.29 (15)	C11—C10—C9	121.33 (19)
C8—N1—C14	119.33 (14)	C11—C10—H10A	119.3
O3—N2—O4	122.38 (17)	C9—C10—H10A	119.3
O3—N2—C16	119.38 (16)	C10—C11—C12	119.9 (2)
O4—N2—C16	118.24 (18)	C10—C11—H11A	120.1
C4—O1—C1	107.33 (19)	C12—C11—H11A	120.1
C2—C1—O1	110.6 (2)	C13—C12—C11	119.8 (2)
C2—C1—H1A	124.7	C13—C12—H12A	120.1
O1—C1—H1A	124.7	C11—C12—H12A	120.1
C1—C2—C3	106.1 (2)	C12—C13—C8	120.85 (19)
C1—C2—H2A	127.0	C12—C13—H13A	119.6
C3—C2—H2A	127.0	C8—C13—H13A	119.6
C4—C3—C2	107.9 (2)	N1—C14—C15	114.53 (15)
C4—C3—H3A	126.0	N1—C14—H14A	108.6
C2—C3—H3A	126.0	C15—C14—H14A	108.6
C3—C4—O1	108.01 (19)	N1—C14—H14B	108.6
C3—C4—C5	135.4 (2)	C15—C14—H14B	108.6
O1—C4—C5	116.57 (17)	H14A—C14—H14B	107.6
C4—C5—C6	111.12 (15)	C20—C15—C16	115.38 (17)
C4—C5—S1	112.40 (14)	C20—C15—C14	120.58 (16)
C6—C5—S1	111.43 (13)	C16—C15—C14	124.03 (16)
C4—C5—H5A	107.2	C17—C16—C15	122.67 (17)
C6—C5—H5A	107.2	C17—C16—N2	115.49 (17)
S1—C5—H5A	107.2	C15—C16—N2	121.83 (17)
C7—C6—C5	112.73 (15)	C18—C17—C16	119.67 (18)
C7—C6—H6A	109.0	C18—C17—H17A	120.2
C5—C6—H6A	109.0	C16—C17—H17A	120.2
C7—C6—H6B	109.0	C19—C18—C17	119.42 (19)
C5—C6—H6B	109.0	C19—C18—H18A	120.3
H6A—C6—H6B	107.8	C17—C18—H18A	120.3
O2—C7—N1	120.66 (17)	C18—C19—C20	120.44 (19)
O2—C7—C6	121.97 (17)	C18—C19—H19A	119.8
N1—C7—C6	117.35 (17)	C20—C19—H19A	119.8
C13—C8—C9	119.62 (17)	C19—C20—C15	122.40 (18)
C13—C8—N1	119.29 (16)	C19—C20—H20A	118.8
C9—C8—N1	121.09 (16)	C15—C20—H20A	118.8
C10—C9—C8	118.34 (18)		

### Hydrogen-bond geometry (Å, °)

**Table:**

*D*—H···*A*	*D*—H	H···*A*	*D*···*A*	*D*—H···*A*
C2—H2A···O2^i^	0.93	2.49	3.401 (3)	168
C12—H12A···O2^ii^	0.93	2.59	3.381 (3)	144

Symmetry codes: (i) −*x*+5/2, *y*−1/2, −*z*+1/2; (ii) *x*, *y*−1, *z*.
